# Status of Government-Funded Radiotherapy Services in Nigeria

**DOI:** 10.1200/GO.22.00406

**Published:** 2023-06-22

**Authors:** Simeon C. Aruah, Runcie C.W. Chidebe, Tochukwu C. Orjiakor, Fatima Uba, Uchechukwu N. Shagaya, Charles Ugwanyi, Aisha A. Umar, Taofeeq Ige, Obinna C. Asogwa, Oiza T. Ahmadu, Musa Ali-Gombe, Alabi Adewumi, Vitalis C. Okwor, Jimoh A. Mutiu, Basheer Bello, Lucy O. Eriba, Yusuf A. Ahmed, Awwal Bisalla, Ukamaka Itanyi, Ramatallah A. Balogun, Suleiman Alabi, David Pistenmaa, C. Norman Coleman, Dosanjh Manjit

**Affiliations:** ^1^ Radiation Oncology Department, National Hospital Abuja, Abuja, Nigeria; ^2^ College of Medicine, University of Abuja, Gwagwalada, Abuja, Nigeria; ^3^ Project PINK BLUE—Health & Psychological Trust Centre, Abuja, Nigeria; ^4^ Department of Sociology & Gerontology, Miami University, Oxford, OH; ^5^ Scripps Gerontology Center, Miami University, Oxford, OH; ^6^ Department of Psychology, University of Nigeria Nsukka, Enugu, Nigeria; ^7^ Radiation and Clinical Oncology Department, National Hospital Abuja, Abuja, Nigeria; ^8^ Neurosurgery Department, National Hospital Abuja, Abuja, Nigeria; ^9^ Department of Radiology, National Hospital Abuja, Abuja, Nigeria; ^10^ Medical Physics Department, National Hospital Abuja, Abuja, Nigeria; ^11^ Radiotherapy Department, Asi Ukpo Comprehensive Cancer Center, Calabar, Nigeria; ^12^ Radiation Oncology Department, Ahmadu Bello University Teaching Hospital (ABUTH), Zaria, Nigeria; ^13^ Radiology Department, Gombe State University, Gombe, Nigeria; ^14^ Department of Radiotherapy and Oncology, Federal Teaching Hospital Gombe, Gombe, Nigeria; ^15^ Radiation Oncology Department, Nigeria Sovereign Investment Authority-Lagos University Teaching Hospital (NSIA-LUTH), Lagos, Nigeria; ^16^ Radiation Oncology Department, University of Nigeria Teaching Hospital (UNTH), Ituku Ozalla, Enugu, Nigeria; ^17^ University of Ibadan/University College Hospital Ibadan, Ibadan, Nigeria; ^18^ Radiation Oncology Department, Usman Danfodio University Teaching Hospital (UDUTH), Sokoto, Nigeria; ^19^ Radiation Oncology Department, University of Benin Teaching Hospital (UBTH), Edo State, Nigeria; ^20^ Centre for Energy Research and Training, Ahmadu Bello University, Zaria, Nigeria; ^21^ Nigeria Atomic Energy Commission (NAEC), Abuja, Nigeria; ^22^ Radiology Department, University of Abuja Teaching Hospital (UATH), Gwagwalada, Abuja, Nigeria; ^23^ University of Abuja Teaching Hosiptal (UATH), Gwagwalada, Abuja, Nigeria; ^24^ International Cancer Expert Corps (ICEC) Inc, Washington, DC; ^25^ University of Oxford, Oxford, United Kingdom; ^26^ European Centre for Nuclear Research (CERN), Geneva, Switzerland

## Abstract

**PURPOSE:**

Access to radiotherapy (RT) is now one of the stark examples of global cancer inequities. More than 800,000 new cancer cases require potentially curative or palliative RT services in Africa, arguably <15% of these patients currently have access to this important service. For a population of more than 206 million, Nigeria requires a minimum of 280 RT machines for the increasing number of cancer cases. Painfully, the country has only eight Government-funded RT machines. This study aimed to evaluate the status of the eight Government-funded RT services in Nigeria and their ability to deliver effective RT to their patients.

**METHODS:**

A survey addressing 10 critical areas was used to assess the eight Government-funded RT services in Nigeria.

**RESULTS:**

Unfortunately, six of the eight centers (75%) surveyed have not treated patients with RT because they do not have functioning teletherapy machines in 2021. Only two RT centers have the capability of treating patients using advanced RT techniques. There is no positron emission tomography-computed tomography scan in any of the Government-funded RT centers. The workforce capacity and infrastructure across the eight centers are limited. All of the centers lack residency training programs for medical physicists and radiation therapy technologists resulting in very few well-trained staff.

**CONCLUSION:**

As the Nigerian Government plans for the new National Cancer Control Plan, there is an urgent need to scale up access to RT by upgrading the RT equipment, workforce, and infrastructure to meet the current needs of Nigerian patients with cancer. Although the shortfall is apparent from a variety of RT-capacity databases, this detailed analysis provides essential information for an implementation plan involving solutions from within Nigeria and with global partners.

## Teaser

Nigeria has the highest burden of cancer in SSA. What is the state of access to radiotherapy in this population?

## INTRODUCTION

Radiotherapy (RT) is an essential component of the curative and palliative treatment of patients with cancer. However, this important modality is not readily available in many African countries including Nigeria.^1^ More than 800,000 new cancer cases require potentially curative or palliative RT services in Africa, arguably <15% of these patients currently have access to this important service.^2,3^ With 125,000 new cancer cases and 79,000 cancer death in 2020^4^ in Nigeria, cancer is becoming a growing public health challenge for its population of 206 million people.^5^ The four most common cancers were breast—22.7%, prostate—12.3%, colorectal—6%, non-Hodgkin lymphoma—5.9%, and other cancers—53.1%^4^ while the most common cancers treated with RT were breast (37.5%), uterine cervix (16.3%), head and neck (11.9%), and prostate (10.9%).^6^

**CONTEXT**

**Key Objective**
This study evaluated the status of Government-funded radiotherapy (RT) services in Nigeria regarding equipment and workforce and quantified their ability to deliver effective RT to patients.
**Knowledge Generated**
Only two Government-funded RT centers have modern three-dimensional computed tomography (CT) simulation equipment.
  There are limited service contracts or maintenance engineers to repair linear accelerators (LINACs) in Nigeria. Thus, when a LINAC breaks down, there is a long waiting time for maintenance which, in turn, leads to treatment delay.
  There is no positron emission tomography-CT scan in any of the Government-funded RT centers.
  There is a lack of residency training programs for medical physicists and radiation therapy technologists in Nigeria.
**Relevance**
This study provided empirical evidence of the poor state of Government-funded RT centers and the need for more budgetary allocation.
  The findings will guide clinicians, patient advocates, health care leaders, and the Nigeria Federal Ministry of Health as they plan for the new National Cancer Control Plan.

Access to RT is a significant challenge globally because of deficiencies in equipment and RT workforce.^7^ In addition to workforce shortages, another critical challenge in delivering quality RT services in Nigeria is poor RT infrastructure. This makes transition from the two-dimensional (2D) to three-dimensional (3D) RT treatment technique difficult in most of the Government-established RT services.^6^ There is also a lack of Government commitment especially in terms of budget allocation, budget releases, monitoring, and implementation of financing for RT infrastructure. The current widely accepted standard for a basic RT center includes at least two teletherapy units, a high-dose rate (HDR) after-loading brachytherapy system, a treatment planning system (TPS), a mould room, a computed tomography (CT) simulator, adequate dosimetry, and quality assurance equipment.^8^ Arguably, there is no Government-funded RT center in Nigeria that meets this standard. Presently, most of the Government-funded centers have yet to experience an upgrade of their RT services as seen in other African countries such as South Africa and Egypt.

The Nigeria Federal Ministry of Health established the National Cancer Control Program (NCCP) in 2006 to develop, coordinate, and implement activities, policies, and programs to address the trend of increasing cancers in Nigeria.^9^ To achieve its goals, the NCCP launched a National Cancer Control Plan for 2008-2013 and another plan for 2018-2022 with priorities for treatment and for hospice and palliative care as goals 2 and 3, respectively. The objectives of goal 2 were to increase by at least 50% the functionality of the comprehensive cancer care centers (which includes RT) and to increase the workforce by 60% by the year 2022 with a budget of N59,508,662.50 ($188,916.39 [USD]).^10^ However, such an important national policy has not taken a firm stand in addressing the challenge of access to cancer care, especially RT services. Arguably, RT is one of the most important parts of a cancer control plan, and its availability to patients globally is quite desirable.^11^ It is also a critical and cost-effective component of a comprehensive cancer control plan that offers the potential for cure, control, and palliation of disease in >50% of patients with cancer.^12,13^ Yet, the disparities in RT access are one of the largest technology gaps in health care today with many patients with cancer lacking access to RT.

To the best of our knowledge, many of the studies published on the status of RT in Nigeria have not taken a close look at the status of the existing Government-funded RT services. There is a lack of holistic assessment of the status of RT machines, workforce, and other elements of the RT infrastructure for centers in Nigeria. To update this information, we have surveyed the current status of RT machines, workforce, and infrastructure in the eight Government-funded RT centers in Nigeria.

## METHODS

**FIG 1:**
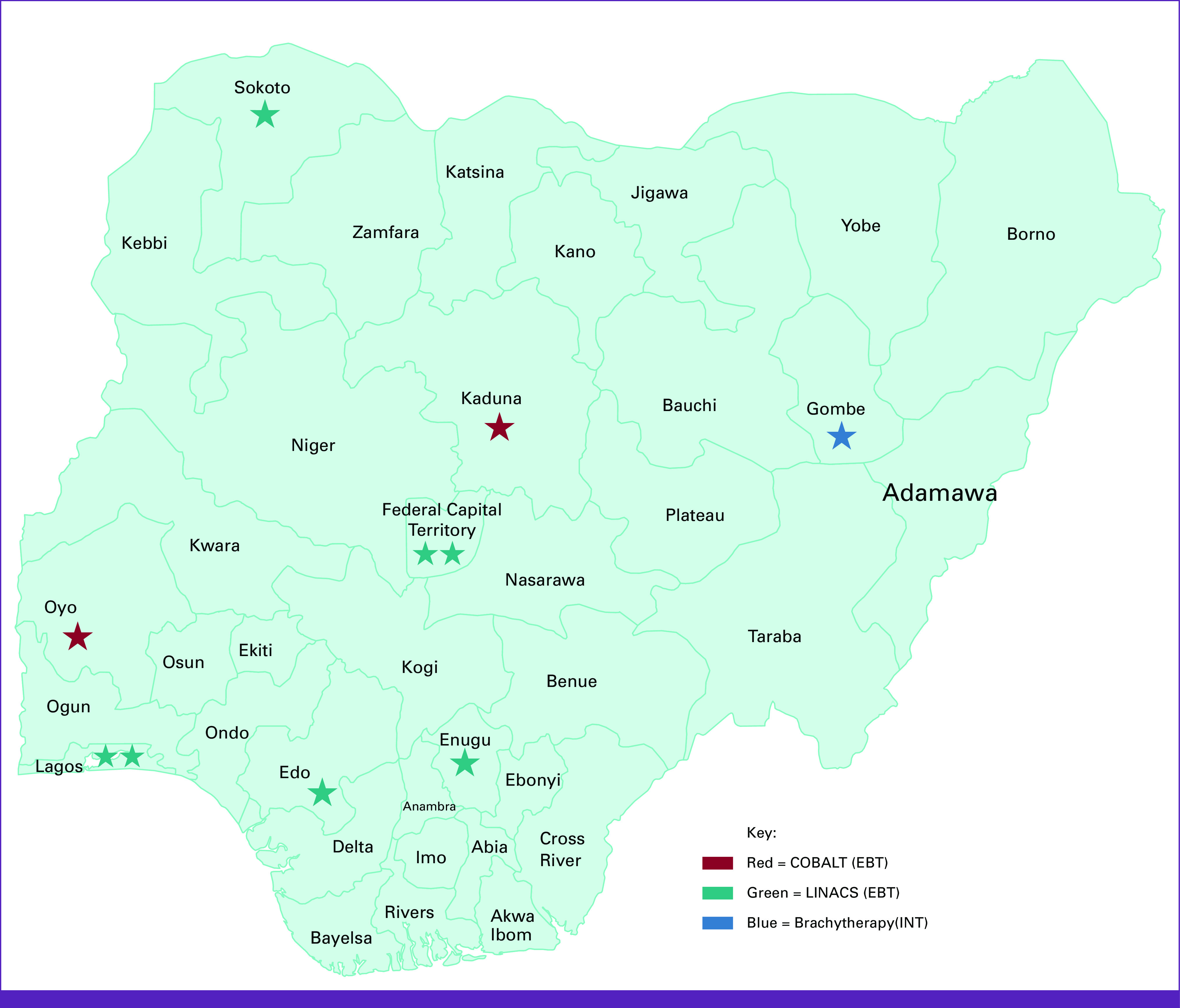
Map showing the location of the Government-funded radiotherapy centers located in all the six geopolitical zones of Nigeria. EBT, External Beam Therapy; INT, Intracavitary Brachytherapy for Gynaecological Cancers; LINACS, linear accelerators

This was a multicenter study of the eight Government-funded RT centers encompassing all six geopolitical zones of Nigeria as of 2021. The RT centers surveyed are the National Hospital Abuja (NHA), the Usman Danfodio University Teaching Hospital (UDUTH) Sokoto, the Ahmadu Bello University Teaching Hospital (ABUTH) Zaria, the University of Nigeria Teaching Hospital (UNTH) Ituku Ozalla—the Enugu, Nigeria Sovereign Investment Authority—Lagos University Teaching Hospital (NSIA-LUTH) Lagos, the Federal Teaching Hospital (FTH) Gombe, the University College Hospital (UCH) Ibadan, and the University of Benin Teaching Hospital (UBTH) Benin (Fig 1). The study involved multistage sampling of services in the RT centers. A survey tool was developed by the investigators to assess 10 critical areas pertaining to the status of RT machines, the RT workforce, and the RT infrastructure in the eight centers. A pilot study of the survey tool was conducted in ABUTH, UNTH, and LUTH to ascertain the feasibility of a national study. After the analysis of the results of the pilot study, the tool was expanded with the addition of items assessing electron energies and diagnostic radiology support. The survey tool was completed by radiation oncologists and medical physicists in the centers in 2021. Six surveys were completed online while two were completed in paper copies. The collected data were double-checked, cleaned, and analyzed using descriptive statistics. The results of the pilot study are not included in the results of the main study.

## RESULTS

### Oncology Workforce in the Radiotherapy Centers

Our results showed that Nigeria has 44 radiation oncologists, 45 radiation oncology residents, 44 medical physicists, eight dosimetrists, 42 radiation therapy technologists (RTTs), 57 oncology nurses, seven mould room technicians, 15 biomedical engineers, and eight nuclear medical physicians working in the Government-funded RT centers. None of the RT centers has a medical physics residency training program (Table 1). Six of the eight RT centers have radiation oncology residency programs, the exceptions being FTH and UBTH. Seventy-five (75%) of the Government-funded RT centers do not have a designated dosimetrist including UNTH Enugu, FTH, NHA, ABUTH, NSIA-LUTH, and UCH. There were no nuclear medicine physicians in FTH and UBTH. There is no mould room technician in FTH, NSIA-LUTH and UCH. NHA has the highest number of oncology nurses. There was no input from UCH regarding the number of oncology nurses.

**TABLE 1:**
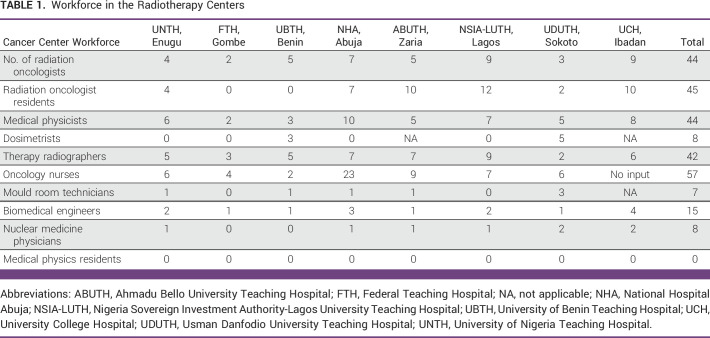
Workforce in the Radiotherapy Centers

### Technologies Available in the Radiotherapy Centers

#### Number of Teletherapy Machines

Only five of the eight RT centers have linear accelerators (LINACs) namely NHA, NSIA-LUTH, UDUTH, UBTH, and UNTH. Only three (60%) of the five centers have functional LINACs, namely UNTH, NHA, and NSIA-LUTH. Thirty percent of the LINACs have broken down. Two centers, UCH and ABUTH, have old unused cobalt-60 teletherapy machines. Four (50%) of the Government-funded RT centers (UBTH, ABUTH, UDUTH, and UCH) do not have a functional teletherapy machine. FTH has neither a cobalt-60 machine nor a LINAC. In total, in the Government-funded RT centers in Nigeria, there are nine teletherapy machines, seven LINACs, and two cobalt-60 machines (Table 2 and Fig 1).

**TABLE 2:**
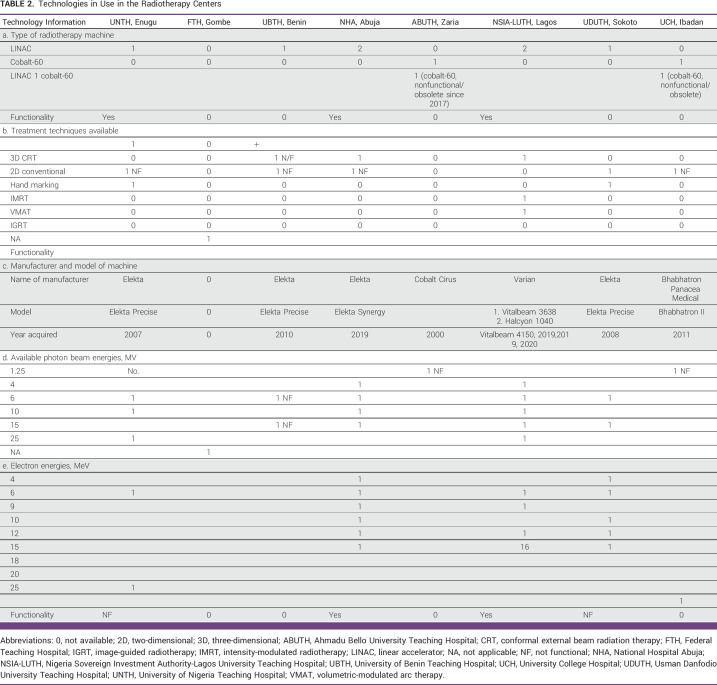
Technologies in Use in the Radiotherapy Centers

#### Energy of photons

Only three of the eight RT centers, UNTH, NHA, and NSIA-LUTH, have multienergy photon LINACs. Having only monoenergy LINACs limits treatment capability.

#### Electron Therapy

Only NHA and NSIA-LUTH have electron capability for treatment of superficial cancers and benign skin diseases. Six (75%) of the Government-funded RT centers cannot treat superficial tumors adequately because they lack the necessary treatment machine capabilities.

### Availability of Brachytherapy Machines

Although all the RT centers have brachytherapy machines, only UCH, FTH, and UBTH have functional HDR brachytherapy machines. Five (63%) of the RT centers with brachytherapy have yet to migrate from 2D to 3D brachytherapy because of lack of CT or magnetic resonance imaging (MRI) simulators which are necessary for image acquisition for their TPSs (Table 3 and Fig 1).

**TABLE 3:**
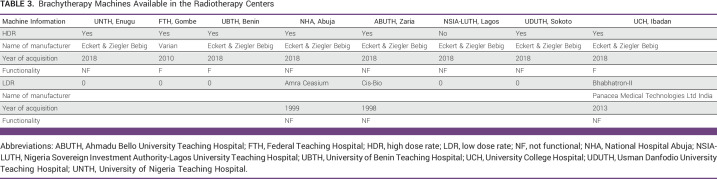
Brachytherapy Machines Available in the Radiotherapy Centers

### Radiotherapy Treatment Planning

#### Availability of Simulation Equipment in the RT Centers

Only NHA and NSIA-LUTH have modern 3D CT simulation equipment, a requirement for modern RT treatment planning. Other centers, UNTH, UBTH, UCH, UDUTH, FTH, and ABUTH, have only 2D conventional simulators using both static and fluoroscopic modes, which are typically limited. Without a CT simulator, it is impossible to deliver a 3D treatment.^14^ The latter technique allows more precise treatment, dose escalation, and reduction of radiation to organ at risk (Table 4).

**TABLE 4:**
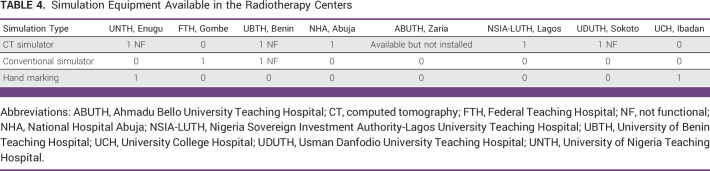
Simulation Equipment Available in the Radiotherapy Centers

#### Availability of TPS

Computerized TPSs are used in teletherapy to generate precise external beam radiation therapy dose distributions that deliver tumoricidal doses to the tumor while sparing normal organs at risk. This important RT system is only available in NHA and NSIA-LUTH. UDUTH has a PRECISE TPS, but it is not functional. UCH has a TPS for brachytherapy (HDR). Four (50%) of the RT centers (UNTH, FTH, UBTH, and ABUTH) have no TPS. Thus, it is possible less accurate dose calculations could compromise treatment delivery (Table 5).

**TABLE 5:**
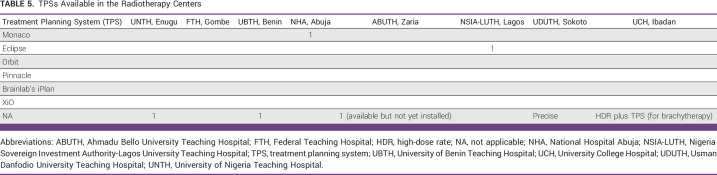
TPSs Available in the Radiotherapy Centers

#### 3D Conformal RT Capacity

Of the eight RT centers, only two centers, NHA and NSIA-LUTH, can provide 3D conformal RT services. Only NSIA-LUTH has the capacity to provide advanced techniques such as volumetric-modulated arc therapy and intensity-modulated RT (IMRT). Thus, six (75%) of the RT centers do not have the capacity to deliver modern RT treatments.

#### Availability of Record and Verify Systems

Record and verify (R&V) systems were developed to reduce the risk of treatment errors in radiation oncology. These have evolved into complete RT information management systems integrating treatment planning computers and treatment delivery systems. Only two centers, NHA and NSIA-LUTH, have R&V systems. NHA uses the Mosaiq system while NSIA-LUTH uses ARIA oncology information system. Six (75%) of the RT centers (UNTH, UDUTH, UCH, FTH, UBTH, and ABUTH) do not have R&V systems. Hence, they are more likely to experience errors in their treatment of patients with cancer.

### Diagnostic Imaging Resources

#### Availability of radiological imaging support

Radiological imaging is critical in making cancer diagnoses, for treatment planning, and for follow-up to assess the results of curative and palliative RT. All the centers surveyed have functional ultrasound, x-ray, and fluoroscopy machines. All except UNTH have functional CT scanners. MRI is available only in NHA, FTH, NSIA-LUTH, and UDUTH. There is no MRI in UNTH, ABUTH, nor UCH. Our results show that most of the radiology imaging machines in the RT centers are not linked by digital communication in medicine (Table 6).

**TABLE 6:**
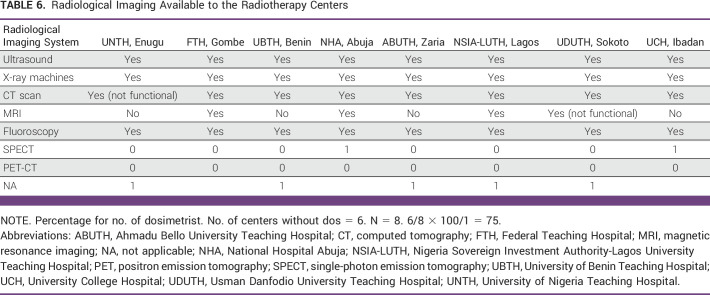
Radiological Imaging Available to the Radiotherapy Centers

#### Availability of radionuclide scans

Only NHA and UCH have single photon emission tomography (SPECT) machines although the current imaging of choice is with positron emission tomography (PET)-CT scanners because of their molecular characterization of cancer. None of the Government-funded RT centers have a PET-CT scanner, hence making diagnoses and follow-up of treatment challenging in those RT centers (Table 6).

### Number of Patients Treated With RT and Their Cancer Types in the Past 2 Years

As of 2021, only two centers, NHA and NSIA-LUTH, have been consistently treating patients with cancer with teletherapy machines. FTH has only a functional HDR brachytherapy machine for the treatment of gynecological malignancies such as cervical, vaginal, and endometrial cancers. In both NHA and NSIA-LUTH, the most common cancer treated by teletherapy was breast cancer. Because of frequent downtime and treatment delays, UNTH Enugu has been inconsistent in treating patients. According to the hospital-based cancer registries at five (63%) of the RT centers (UBTH, UCH, ABUTH, UDUTH, and FTH), they have not treated patients with cancer with RT in most of 2021 (Table 7).

**TABLE 7:**
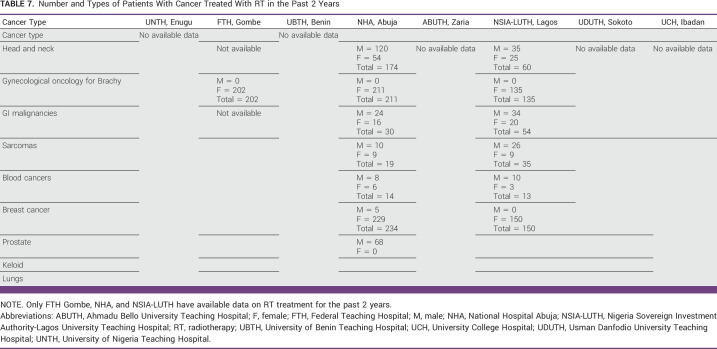
Number and Types of Patients With Cancer Treated With RT in the Past 2 Years

### Estimated Cost of Radiotherapy Treatment in the Government-Funded RT Centers

Only FTH, NHA, and NSIA-LUTH reported cost estimates for both curative and palliative treatments. FTH Gombe presented costs for only gynecological treatments. NSIA-LUTH is a public-private partnership (PPP) RT center with higher cost of treatment compared with NHA which is completely funded by the Government and whose services are provided at subsidized rates. There is no price uniformity for teletherapy services hence making affordability a huge challenge for much of the population. There were no available records from UNTH although it is being operated as PPP. Four (50%) of the RT centers (UBTH, ABUTH, UDUTH, and UCH) have no records on the cost of RT services as these centers have not been treating patients.

## DISCUSSION

Across all the Government-funded RT centers in Nigeria, there is an overall picture of poor workforce strength relative to the number of patients with cancer being managed at the various centers. This has resulted in high clinical workload, treatment delays, and long waiting times to start treatment. Our results show that Nigeria lacks well-structured training programs for the essential oncology workforce for RT delivery such as those for medical physicists, RTTs, and biomedical engineers. Some RT centers lack radiation oncology residency training programs necessitating their sending trainees to other RT centers. Our results suggest that there is an urgent need for RT workforce training and continuing education which would be of great benefit to patients. This is in line with a previous study which identified that expert knowledge has an impact on quality of patient care at all levels.^15^ A radiation oncology center should have at least a teletherapy machine, a radiation oncologist, a medical physicist, and two RTTs per 250,000 people.^8,16^ Our results showed that there is no Government-funded RT center in Nigeria that has all the recommended workforce needed for RT services based on that International Atomic Energy Agency recommendation. Clearly, in Nigeria, there is shortage of necessary workforce for effective RT service delivery.

Our reported findings depict an abysmal state of access to RT services in Nigeria. As of 2021, only two Government-funded RT centers, NHA and NSIA-LUTH, were delivering teletherapy services using LINACs. Most of the Government-funded RT centers in Nigeria do not have a service contract or maintenance engineers to repair LINACs. Thus, when a LINAC breaks down, there is a long waiting time for maintenance which, in turn, leads to treatment delay. Our results agree with reports from other investigators.^6,17-19^ Our findings also showed that Nigeria has a total of nine Government-funded teletherapy machines, seven LINACs, and two cobalt-60 machines. However, maintaining functionality of these RT machines is a huge challenge. NHA and NSIA-LUTH have LINACs with multienergy photons and electrons, 3D conformal RT capability, modern CT simulators, TPSs, and R&V systems for optimal RT service delivery. Although NHA and NSIA-LUTH are the most modern Government-funded RT centers in Nigeria, the other 75% of the Government-funded RT centers do not have the capacity or capability to deliver RT with modern techniques. Our findings are consistent with previous studies.^6,20^ Although we found that there were no working cobalt-60 as of 2021, a study found that all cobalt-60s were in clinical use in 2020 and that all four cobalt-60 machines in Nigeria are Government-funded.^21^ Our finding further confirms the frequent breakdown of RT machines in Nigeria. On the other hand, we argue that only two cobalt-60 machines are Government-funded in Nigeria.

RT involves potential risks because even a small error in treatment planning (dosimetry) or treatment can lead to significant negative consequences. These potential risks are magnified in the use of modern radiation therapy techniques such as IMRT, image-guided RT, and stereotactic radiosurgery that represent an entirely new paradigm that also requires extensive knowledge and understanding of the latest imaging systems, set up uncertainties, radiobiological response of healthy tissues, 3D dose calculations, optimization of variable intensity beam delivery, and internal organ motion.^22^ More than 75% of the RT centers in our survey lacked all the necessary equipment to deliver treatment with complex advanced RT techniques. Our findings are supported by other studies.^16,18^

Our survey showed that Nigerian patients with cancer have limited access to radionuclide scans. Only two centers offer SPECT services, but they rely on importation of the radiopharmaceuticals from overseas. There is no PET-CT scanner in the Government-funded RT centers. This lack of availability of modern imaging for the diagnosis of cancer and for RT treatment planning adversely affects treatment outcomes, including survival, has been reported by several researchers.^23,24^ As of the time of writing this report, MeCure Healthcare, a private center, started operating a PET/CT scanner in Lagos, Nigeria (September 2021). However, major barriers to its use are the cost of the procedure and a lack of awareness by the public of its importance.

Our survey highlights the total number and the most common types of patients with cancer treated with teletherapy machines in the past 2 years in the Government-funded RT centers in Nigeria. Our results show that breast cancer is the most common cancer treated in the functional RT centers. Our results are in congruity with GLOBOCAN and other studies whose findings showed that breast cancer is among the five most common cancers seen in Nigerian cancer centers.^4^

Our study confirmed nonuniformity in the cost of RT services in Nigeria. For instance, in NHA the cost of RT ranges from $760 in US dollars (USD) for palliative treatment to $1,304 (USD) for radical treatment. While in NSIA-LUTH, which is PPP RT center, the cost of RT ranges from $1,300 (USD) for palliative treatment to $2,200 (USD) for radical treatment. That such a price variation exists between Government-funded and PPP RT centers was supported by a previous study that found that the average cost of a 20-fraction course of external beam RT (EBRT) using PPP RT equipment was $1,810 (USD), which represents a 335% increase when compared with EBRT in Government-funded RT facilities where the average cost is $416 (USD).^21^ Our findings were further strengthened by reports from other studies.^25^ The high cost of RT treatment has been a major challenge for patients in accessing RT treatment when the treatment machines are operational, a status often jeopardized by frequent breakdown of RT machines.^22^ To improve access to RT, we recommend that the cost of RT services be subsidized in Government-funded and PPP RT centers, and there is a need for a new model where availability and affordability needs can be met while maintaining high standard of care.

Part of the limitations of this study was noninclusion of private RT centers. We are seeking funds to survey the private RT centers. In addition, some of the data are likely to change on the basis of RT workforce migration and installation of new HDR brachytherapy centers that were not captured in our report. Poor record keeping by hospital-based cancer registries has influenced the accuracy of patient numbers. The use of only quantitative research methodology is also a limitation. An exploratory qualitative data set can match the quantitative data with robust meaning to the challenges and generate sustainable solutions. Hence, future studies should consider using mixed-method research approach.

In conclusion, Nigerian RT services are in crisis mode as noted in this study. However, there is great potential to improve these services in this country of 206 million people. Therefore, there is a need to scale up the number of medical LINACs, improve workforce capacity through training and education programs, and strengthen basic infrastructure in all the Government-funded RT centers using national resources, donors, nongovernmental organizations' support, PPP,^21^ and other private investment. RT is a critical component of a comprehensive national cancer control plan and health investment. We hope that our findings will stimulate increased budget allocations in the future for health care in general and especially for RT centers to bring the best RT services possible to patients with cancer in Nigeria.

## References

[1] Ndlovu N Radiotherapy treatment in cancer control and its important role in Africa Ecancermedicalscience 13 942 2019 31552115 10.3332/ecancer.2019.942 PMC6722105

[2] Rawlinson FM Gwyther L Kiyange F et al The current situation in education and training of health-care professionals across Africa to optimise the delivery of palliative care for cancer patients Ecancermedicalscience 8 492 2014 25624873 10.3332/ecancer.2014.492 PMC4303614

[3] Yennurajalingam S Amos CE Jr Weru J et al Extension for community healthcare outcomes-palliative care in Africa program: Improving access to quality palliative care J Glob Oncol 5 1 8 2019 10.1200/JGO.19.00128 PMC6776016 31335237

[4] International Agency for Research on Cancer (IARC) The Global Cancer Observatory (GCO): Nigeria, 2020 https://gco.iarc.fr/today/data/factsheets/populations/566-nigeria-fact-sheets.pdf

[5] National Population Commission (NPC) National Policy on Population for Sustainable Development. NPC Publication 1st ed., Abuja, Nigeria, 2021 https://drive.google.com/file/d/1_LqDbc249sq_bo_Cmpa8VSZBmk8fHJSj/view?usp=embed_facebook

[6] Leng J Ntekim AI Ibraheem A et al Infrastructural challenges lead to delay of curative radiotherapy in Nigeria JCO Glob Oncol 10.1200/JGO.19.00286 2020 10.1200/JGO.19.00286 PMC7051797 32083951

[7] Zubizarreta EH Fidarova E Healy B et al Need for radiotherapy in low and middle income countries—The silent crisis continues Clin Oncol (R Coll Radiol) 27 107 114 2015 25455407 10.1016/j.clon.2014.10.006

[8] Balogun O Rodin D Ngwa W et al Challenges and prospects for providing radiation oncology services in Africa Semin Radiat Oncol 27 184 188 2017 28325246 10.1016/j.semradonc.2016.11.011 PMC5653208

[9] Federal Ministry of Health National Cancer Control Programme 2023. https://www.health.gov.ng/index.php?option=com_content&view=article&id=329&Itemid=551

[10] Federal Ministry of Health National Cancer Control Plan 2018-2022 2018. https://www.iccp-portal.org/system/files/plans/NCCP_Final%20%5B1%5D.pdf

[11] Rodin D Abdel-Wahab M Lievens Y Global radiotherapy challenge: Turning data into action Lancet Glob Health 6 S15 S16 2018

[12] Abdel-Wahab M Bourque JM Pynda Y et al Status of radiotherapy resources in Africa: An International Atomic Energy Agency analysis Lancet Oncol 14 e168 e175 2013 23561748 10.1016/S1470-2045(12)70532-6

[13] Barton MB Gebski V Manderson C et al Radiation therapy: Are we getting value for money? Clin Oncol 7 287 292 1995 10.1016/s0936-6555(05)80535-7 8580053

[14] Vatnitsky S Rosenblatt E Transition From 2-D Radiotherapy to 3-D Conformal and Intensity Modulated Radiotherapy Vienna International Atomic Energy Agency 2008

[15] Martin MG Chidebe RCW Nwaneri MO et al Impact of 10-day Fulbright Specialist Program and Project Pink Blue Education Sessions on medical oncology knowledge among physicians who treat cancer in Nigeria J Cancer Educ 38 378 382 2022 35838882 10.1007/s13187-021-02130-y

[16] International Atomic Energy Agency (IAEA) Division for Human Health: DIRAC (DIrectory of RAdiotherapy Centres) 2022. https://dirac.iaea.org/Query/Countries

[17] Ige TA Jenkins A Burt G et al Surveying the challenges to improve linear accelerator-based radiation therapy in Africa: A unique collaborative platform of all 28 African countries offering such treatment Clin Oncol (R Coll Radiol) 33 e521 e529 2021 34116903 10.1016/j.clon.2021.05.008

[18] Fatunmbi M Saunders A Chugani B et al Cancer registration in resource-limited environments-experience in Lagos, Nigeria J Surg Res 235 167 170 2019 30691791 10.1016/j.jss.2018.09.021 PMC11209915

[19] Fatiregun OA Bakare O Ayeni S et al 10-Year mortality pattern among cancer patients in Lagos State University Teaching Hospital, Ikeja, Lagos Front Oncol 10 573036 2020 33330046 10.3389/fonc.2020.573036 PMC7735062

[20] Wroe LM Ige TA Asogwa OC et al Comparative analysis of radiotherapy linear accelerator downtime and failure modes in the UK, Nigeria and Botswana Clin Oncol (R Coll Radiol) 32 e111 e118 2020 31757747 10.1016/j.clon.2019.10.010

[21] Anakwenze Akinfenwa CP Ibraheem A Nwankwo K et al Emerging use of public-private partnerships in public radiotherapy facilities in Nigeria JCO Glob Oncol 10.1200/GO.21.00066 2021 10.1200/GO.21.00066 PMC8389883 34351813

[22] Anacak Y Zubizarreta E Zaghloul M et al The practice of paediatric radiation oncology in low- and middle-income countries: Outcomes of an international atomic energy agency study Clin Oncol (R Coll Radiol) 33 e211 e220 2021 33250288 10.1016/j.clon.2020.11.004

[23] Lou B Doken S Zhuang T et al An image-based deep learning framework for individualising radiotherapy dose: A retrospective analysis of outcome prediction Lancet Digit Health 1 e136 e147 2019 31448366 10.1016/S2589-7500(19)30058-5 PMC6708276

[24] Press RH Shu HKG Shim H et al The use of quantitative imaging in radiation oncology: A quantitative imaging network (QIN) perspective Int J Radiat Oncol Biol Phys 102 1219 1235 2018 29966725 10.1016/j.ijrobp.2018.06.023 PMC6348006

[25] Datta N Rogers S Bodis S Challenges and opportunities to realize "The 2030 Agenda for Sustainable Development" by the United Nations: Implications for radiation therapy infrastructure in low- and middle-income countries Int J Radiat Oncol Biol Phys 105 918 933 2019 31451317 10.1016/j.ijrobp.2019.04.033

